# Midnight punctual tachycardia

**DOI:** 10.1007/s12471-025-01958-z

**Published:** 2025-05-08

**Authors:** Robin Kuijpers, Kim Smulders, Pepijn van der Voort, René Tio, Luuk Otterspoor

**Affiliations:** Department of Cardiology, Catharina Hospital, Eindhoven, The Netherlands

A 75-year-old male presented with an electrical storm of monomorphic ventricular tachycardias (VT) with varying morphologies at 110 beats per minute. He had a history of ischaemic cardiomyopathy following an inferoposterolateral infarction. He underwent CRT‑D implantation in 2016 because of a left ventricular ejection fraction of 30% with left bundle branch block.

Upon admission, we treated him consecutively with amiodarone, procainamide, propranolol, lidocaine, and eventually sedation and intubation [1]. During the day, the patient remained free of arrhythmias. However, at night we observed repetitive nocturnal ventricular tachycardias beginning precisely at midnight and recurring at regular intervals of 30 min, for a total of six episodes, as illustrated in Fig. 1. The onset of this VT is shown in Fig. 2.

**Fig. 1:**
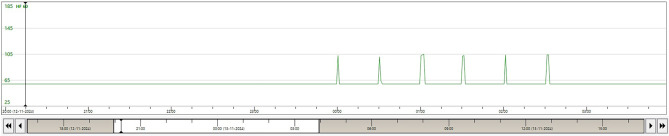
The heart rate graph illustrates a paced rhythm at 60 beats per minute. A repetitive ventricular tachycardia begins precisely at midnight and recurs at 30-minute intervals, culminating in a total of six episodes

**Fig. 2:**
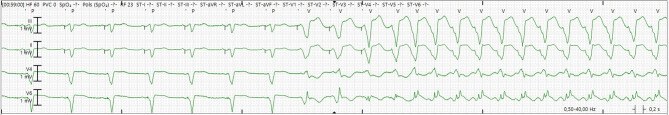
The rhythm strip shows the onset of ventricular tachycardia

What could be the aetiology of this recurring nocturnal arrhythmia?

## Answer

You will find the answer elsewhere in this issue.
